# Senolytic treatment attenuates immune cell infiltration without improving IAV outcomes in aged mice

**DOI:** 10.1111/acel.14437

**Published:** 2025-01-03

**Authors:** Adrian Luna, Kai‐Neng Chou, Kathleen M. Wragg, Matthew J. Worley, Nikhil Paruchuri, Xiaofeng Zhou, Muriel G. Blin, Bethany B. Moore, Morgan Salmon, Daniel R. Goldstein, Jane C. Deng

**Affiliations:** ^1^ Division of Pulmonary and Critical Care Medicine, Department of Internal Medicine University of Michigan Ann Arbor Michigan USA; ^2^ Department of Microbiology and Immunology University of Michigan Ann Arbor Michigan USA; ^3^ Division of Cardiology, Department of Internal Medicine University of Michigan Ann Arbor Michigan USA; ^4^ Department of Cardiac Surgery University of Michigan Ann Arbor Michigan USA; ^5^ Veterans Affairs Ann Arbor Healthcare System Ann Arbor Michigan USA

**Keywords:** aging, cellular senescence, pulmonary host defense, respiratory viral infections, senolytics

## Abstract

Aging is a major risk factor for poor outcomes following respiratory infections. In animal models, the most severe outcomes of respiratory infections in older hosts have been associated with an increased burden of senescent cells that accumulate over time with age and create a hyperinflammatory response. Although studies using coronavirus animal models have demonstrated that removal of senescent cells with senolytics, a class of drugs that selectively kills senescent cells, resulted in reduced lung damage and increased survival, little is known about the role that senescent cells play in the outcome of influenza A viral (IAV) infections in aged mice. Here, we tested if the aged mice survival or weight loss IAV infections could be improved using three different senolytic regimens. We found that neither dasatinib plus quercetin, fisetin, nor ABT‐263 improved outcomes. Furthermore, both dasatanib plus quercetin and fisetin treatments further suppressed immune infiltration than aging alone. Additionally, our data show that the short‐term senolytic agents do not reduce senescent markers in our aged mouse model. These findings suggest that acute senolytic treatments do not universally reverse aging related immune phenotype against all respiratory viral infections.

## Graphical abstract

Aging exacerbates IAV infection severity in aged mice. Senolytic treatment fail to improve IAV morbidity and survival in aged mice. Senolytics suppress immune infiltration into the lungs. Acute senolytic treatment does not reduce senescence markers.

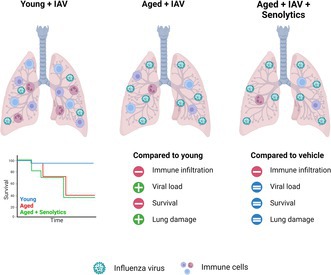

**Abbreviations**
BAL: bronchoalveolar lavage
BALF: bronchoalveolar lavage fluid
BCA: bicinchoninic acid assay
D + Q: dasatanib plus quercetin
EDTA: ethylenediaminetetraacetic acid
gWAT: gonadal white adipose tissue
IAV: influenza A Virus
IHC: immunohistochemistry
MSD: Meso Scale Discovery
PBS: phosphate buffered saline
PBS‐T: phosphate buffered saline with 0.1% tween‐20
PFA: paraformaldehyde
PFU: plaque forming units
PHBTEN: normal primary human bronchial/tracheal epithelial
PVDF: polyvinylidene fluoride
SASP: senescence associated secreted proteins
TPER: tissue protein extraction reagent

## 1 INTRODUCTION

The recent SARS‐CoV‐2 pandemic has highlighted that age is a critical factor in respiratory infections by disproportionally becoming life‐threating in older adults. The majority of deaths from SARS‐CoV‐2 and influenza infections occur in individuals over 65 years of age, accounting for ~76% of COVID‐19 deaths since the beginning of the pandemic and ~83% of IAV deaths for the 2021–2022 season in the United States, respectively (Elflein, 2023a, 2023b). Studies suggest that immunological changes that occur with aging are major contributors to different outcomes between young and older individuals.

Aging has been shown to cause dysregulation of the immune system's innate and adaptive arms (Bartleson et al., 2021; Pietrobon et al., 2020). The innate immune system alterations due to aging are currently characterized by dysfunction of macrophages, neutrophils, and dendritic cells resulting in a reduced ability to recognize and respond to pathogens (Agrawal & Gupta, 2011; De Maeyer & Chambers, 2021; Van Avondt et al., 2023). Aging also induces an altered cytokine response that results in elevated levels of basal inflammation in older individuals, which is thought to contribute to the hyperinflammatory response during respiratory infections that leads to increased lung damage (Camell et al., 2021; Kulkarni et al., 2019). Cumulatively, the changes in the immune response that occur with age promote more severe outcomes to infections.

Previous studies suggest that age‐associated immune alterations are attributable in part to the accumulation of senescent cells. Senescent cells are metabolically active cells that have in most cases permanently exited the cell cycle through expression of p16 and p21, cell cycle regulators (Fang et al., 1999; Krishnamurthy et al., 2004). The senescent cells also adopt a phenotype known as senescence‐associated secretory phenotype (SASP) in which they secrete inflammatory cytokines, chemokines, and extracellular matrix‐degrading enzymes (Kuilman & Peeper, 2009). Although the SASP factors promote wound healing and tumor suppression in normal circumstances, the accumulation of senescent cells due to the aging process creates elevated basal inflammation that promotes decline in physical function and progression of age‐related diseases (Schmitt et al., 2022; Wilkinson & Hardman, 2020; Xu et al., 2018). Removal of senescent cells by genetic means or with a class of drugs called senolytics improves physical function, increases lifespan, and delays onset of aging related diseases such as chronic kidney disease, idiopathic pulmonary fibrosis, and others (Baker et al., 2011; Hambright et al., 2023; Mahoney et al., 2023; Martínez‐Cué & Rueda, 2020; Schafer et al., 2017; Yousefzadeh et al., 2018; Zhu et al., 2015). These findings highlight the potential of senolytics to improve outcomes and delay development of age‐related diseases.

Viral infections can induce senescence acutely (Lee et al., 2021; Tripathi et al., 2021; Tsuji et al., 2022). This phenomenon, called virus‐induced senescence (VIS), is indistinguishable from aging related senescence with both processes leading to cell cycle arrest and adoption of SASP (Lee et al., 2021; Tripathi et al., 2021). In SARS‐CoV‐2 infection models, the induction of VIS cells was associated with worsened lung injury and decreased survival (Lee et al., 2021), suggesting that senescent cells are detrimental for respiratory infections. Prior studies have reported that older rodents (over 20‐month‐old) demonstrate increased morbidity and/or mortality from coronavirus infections (Camell et al., 2021; Delval et al., 2023) and that senolytic treatments, including dasatanib plus quercertin (D + Q), fisetin, and ABT‐263 (navitoclax) in aged rodent models of coronavirus infection have been shown to reduce inflammatory responses and improved outcomes (Camell et al., 2021; Delval et al., 2023; Pastor‐Fernandez et al., 2023). These studies suggest that the removal of senescent cells in older individuals might have therapeutic benefits for respiratory infections.

While the benefits of senolytic treatments in SARS‐CoV‐2 models are well established, their role in combating influenza A virus (IAV) infections remain unclear. Our studies and those of others have shown that aged animals are more susceptible to IAV infections, mirroring the increased severity observed in elderly human patients (Kulkarni et al., 2019; Lefebvre et al., 2016). This increased susceptibility in aged mice has been linked to a higher burden of senescent alveolar epithelial cells in the lungs, suggesting that these senescent cells may contribute to more severe outcomes in older hosts (Chen et al., 2022; Kulkarni et al., 2019). The worsened outcomes may result from the elevated production of TGF‐beta by age‐induced senescent cells, which affects T cell differentiation during influenza infection and leads to an increased proportion of regulatory T cells (Tregs) compared to young mice (Lorenzo et al., 2022). Interestingly, this shift towards Tregs was reversible with D + Q treatment (Lorenzo et al., 2022). However, a recent study found that D + Q treatment does not reduce morbidity from IAV infection in aged hosts nor restore the memory immune response upon a secondary IAV challenge (Torrance et al., 2023). Given the increased burden of senescent cells in the lungs of aged mice and their potential role in worsening IAV outcomes, we sought to determine whether senolytic compounds could improve these outcomes. Therefore, we treated aged mice of 18 months or older with D + Q, fisetin, or ABT‐263, infected them with IAV, and monitored their health outcomes.

## 2 RESULTS

### 2.1 Markers of senescence increase with aging in C57BL/6 mouse model

**FIGURE 1:**
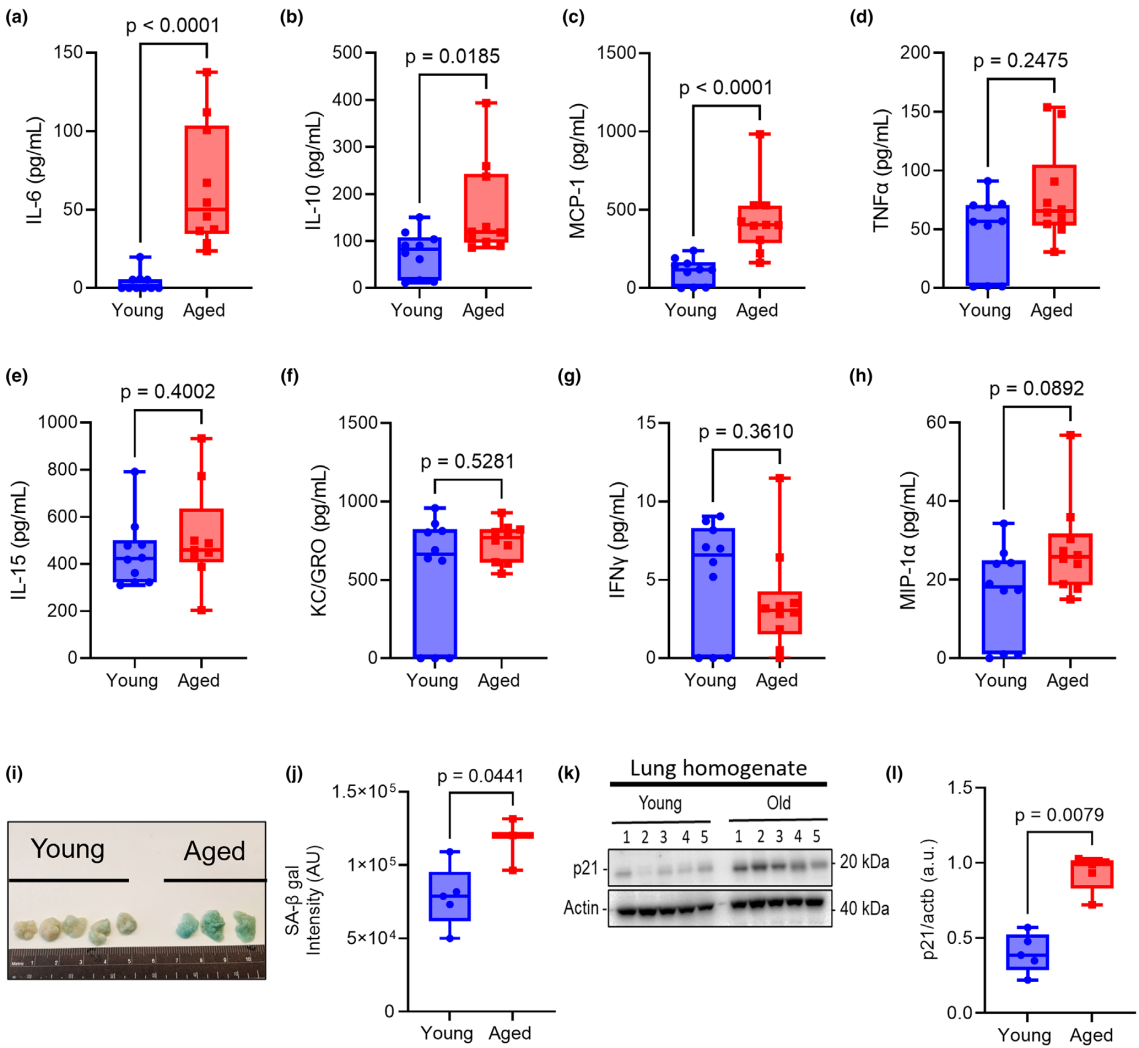
Senescence markers increase with aging in C57BL6/J mice. Serum, adipose and lung tissues were harvested from female young (2–4 months old) and aged (18 months old) C57BL/6J mice and processed for ELISAs or X‐gal staining and immunoblots. (a–h) Serum samples from young and aged mice (*n* = 10 per group) were used on the MSD multi‐spot assay to detect SASP factors. (i) Gonadal white adipose tissue stained with X‐Gal to detect b‐galactosidase activity (*n* = 3–5 per group) (j) Densitometry analysis was performed on stained adipose tissues using Fiji. (k) Total Lung lysates (25 μg/lane; *n* = 5 per group) were analyzed by SDS‐PAGE and immunoblot. (l) Densitometry analysis of lung homogenate immunoblot. Box and whisker plots represent the median and range of the data, which were analyzed by Mann–Whitney test.

We first assessed senescence markers in our C57BL/6 aged murine model (Kulkarni et al., 2019; Smith et al., 2019). Serum from young and aged naïve mice was analyzed for SASP factors by MSD multi‐spot assay (Figure 1a–h). Cytokines IL‐6 (*p* < 0.0001), IL‐10 (*p* = 0.0185), and MCP‐1 (*p* < 0.001) were found to be significantly elevated in aged mice (Figure 1a–c), while TNFα, IL‐15, KC/GRO, IFNγ, and MIP‐1α were unchanged (Figure 1d–h). IL‐1β concentration was below detection limit (LOD >~5 pg/mL). We next measured the expression of β‐Gal activity in the gonadal white adipose tissue (gWAT) as senescent cells have been shown to have upregulated senescence‐associated β‐Gal expression (Dimri et al., 1995). Our staining showed that β‐Gal activity in gWAT was significantly higher in aged mice (*p* = 0.0441) compared to young (Figure 1i,j). As we were interested in the contribution of the senescent cells in the lungs to respiratory infection outcomes, we measured the levels of p21 via Western blot in whole lung homogenates of young and aged mice. We found that p21 was significantly elevated (*p* = 0.0079) in the lung homogenates of aged mice relative to the young samples (Figure 1k,l). These results show that 18‐month‐old C57BL/6 mice show increased markers of senescence in serum, adipose tissue, and lungs compared to young mice.

### 2.2 IAV infection morbidity and mortality increases with age

**FIGURE 2:**
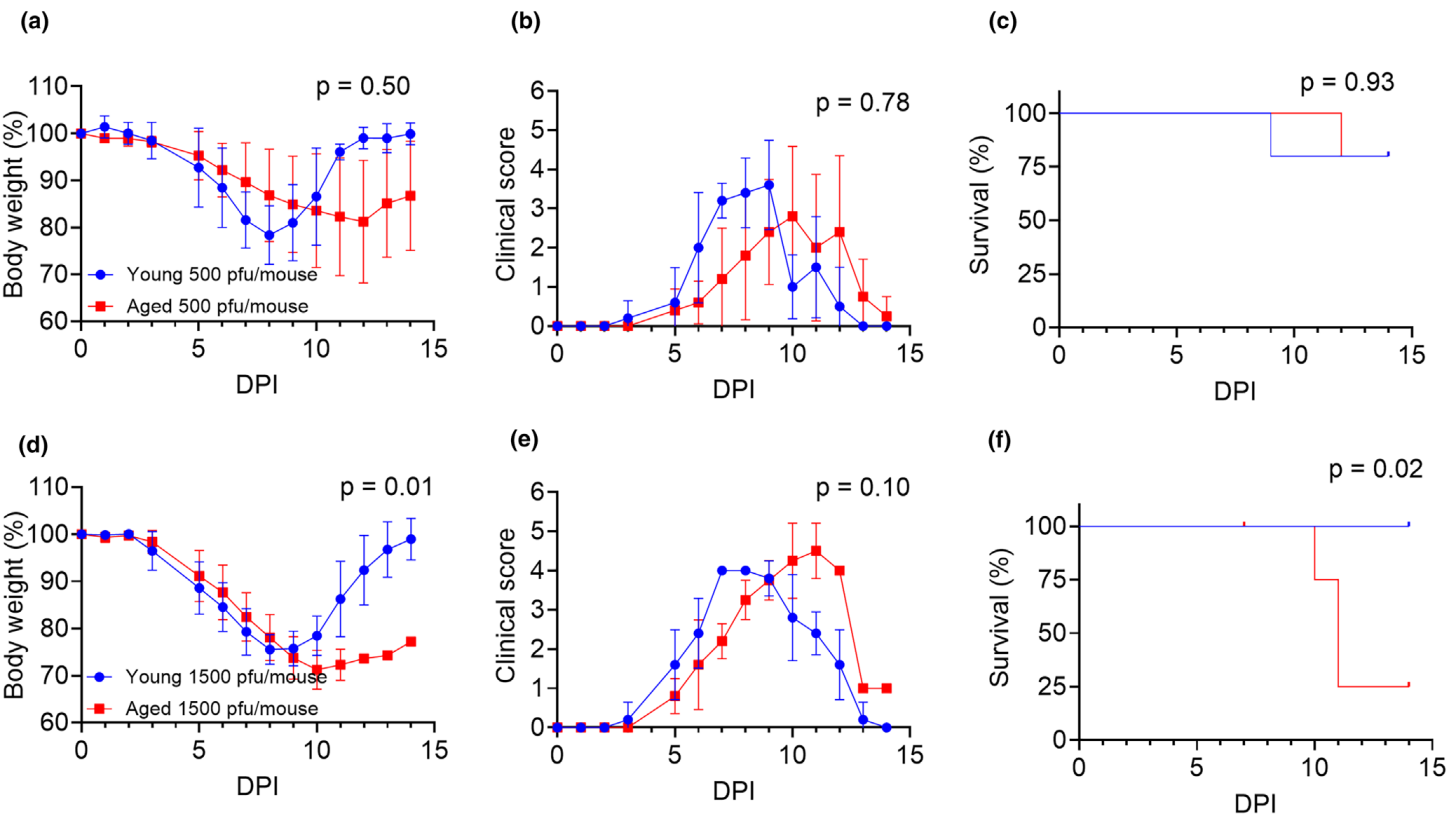
Influenza A infection mortality and morbidity increases with age. Young female (2–4 months old) and aged female (20–21 months old) C57BL/6J mice were infected with 500 PFU/mouse or 1500 PFU/mouse and mice health was tracked daily for 2 weeks (*n* = 5 per group). (a, d) Body weight curve of young and aged mice (*n* = 5 per group). (b, e) Semiquantitative clinical scores. Body weight data and clinical score data were analyzed by Mixed ANOVA. The *p* value represents the effect of age on body weight or clinical score. (c, f) Survival curve comparing young versus aged mice groups analyzed by log‐rank test.

To determine if IAV infection morbidity and mortality worsens with age, we infected young (2–4 months old) and aged mice (18–24 months old) mice with IAV strain A/PR/8/34 intranasally with either 500 PFU/mouse or a 1500 PFU/mouse dose. Weights, clinical scores, and survival were tracked daily for 2 weeks starting from the day of infection (i.e., day 0). At the 500 PFU dose, there were no significant differences in weight loss, clinical scores, or survival between young and old mice (Figure 2a–c), although a slight delay in the onset of severe symptoms and subsequent recovery was noted (Figure 2a,b). At 1500 PFU/mouse, both groups rapidly lost weight between days 4 through 9 (Figure 2d). At day 9 the young mice began to recover, with all mice fully regaining weight by day 14, while the aged mice that survived to day 14 were still ~20% below their initial weight (*p* = 0.01; Figure 2d). Aged mice had higher clinical scores, maintaining them beyond day 9, while young mice peaked at days 8 (Figure 2e). Mortality in aged mice was significantly increased (*p* = 0.0250; Figure 2f). We assessed the viral load of day 8 and 16 post infection by measuring levels of influenza M gene after an intranasal infection of 500 pfu/mouse. Viral load analysis on day 8 and day 16 post‐infection showed a trend toward reduced viral RNA in both groups over time (Figure S1a,b). Aged mice tended to have a higher viral load at day 8 post infection compared to young mice (*p* = 0.0960; Figure S1c). but both effectively cleared the virus by day 16 (Figure S1a–c).

### 2.3 Aged animals display elevated lung injury and decreased lymphocyte responses

**FIGURE 3:**
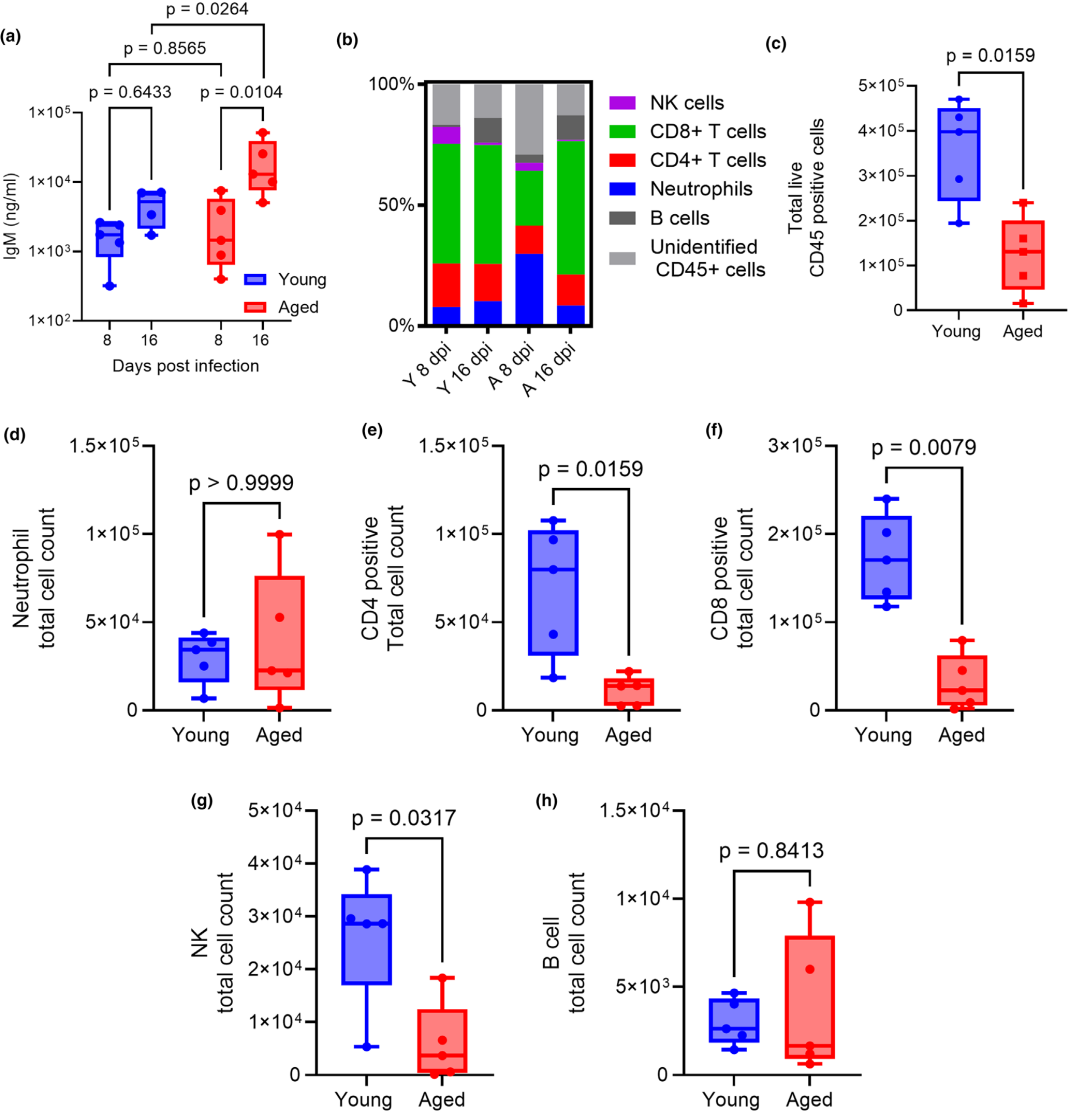
Aged animals display elevated lung injury and decreased lymphocyte responses. (a–h) Young female (2–4 months old) and (20–21 months old) aged female mice were infected with IAV (500 PFU/mouse) and BALF was harvested on day 8 and day 16 post infection to measure IgM and immune cell infiltration (a) Quantification of IgM concentration in the BALF (*n* = 4–5 per group). (b) Immune cell infiltration frequencies out of total CD45 positive cells (*n* = 5 per group). (c) Quantification of total number of live CD45+ cells infiltration in the lung at day 8 post infection (*n* = 5 per group). (d–h) Total cell count of neutrophil, CD4+ T cells, CD8+ T cells, natural killer cells, and B cells (*n* = 5 per group). Box and whisker plots represent the median and range of the data analyzed by Mann–Whitney test.

A major complication of severe influenza infections in older patients is severe lung injury. Thus, we investigated if there were differences in vascular permeability, a surrogate measure of lung damage, as well as immune cell infiltration between young versus aged groups (Figure 3). Following infection of young and aged mice with 500 PFU/mouse, we collected bronchoalveolar lavage fluid (BALF) to measure IgM concentrations and enumeration of immune cells in the BALF by flow cytometry. Although IgM levels in the BALF were similar between the young and aged groups at day 8 post‐infection, we observed markedly elevated IgM levels by day 16 in aged animals compared to day 8 (*p* = 0.0104) and young animals at day 16 (*p* = 0.0264), suggesting increased lung damage compared to young mice (Figure 3a). Examination of immune cell populations at day 8 and 16 identified a relative expansion of neutrophil at day 8 and delays in CD8+ T cell influx in aged animals compared to young animals (Figure 3b). However, the total number of CD45+ cells (*p* = 0.0159), and the absolute number of CD4+ T cells (*p* = 0.0159), CD8+ T cells (*p* = 0.0079), and natural killer cells (*p* = 0.0317) were significantly decreased at day 8 in aged mice compared to young mice (Figure 3c–h), supporting previous reports in aged mice that demonstrated reductions in T cell number during IAV infections (Lu et al., 2018). We found no differences in the frequencies and total cell populations between the young and aged mice (Figure S1a–h) on day 16. These results indicate that aging impairs infiltration of T cells and natural killer cells during the acute phase of IAV infections which is associated with increased vascular permeability during later time points of infection. These results highlight the contribution of age to the immune response and outcomes of IAV infections.

### 2.4 Senolytic pretreatment does not improve outcomes of IAV infections in aged mice

**FIGURE 4:**
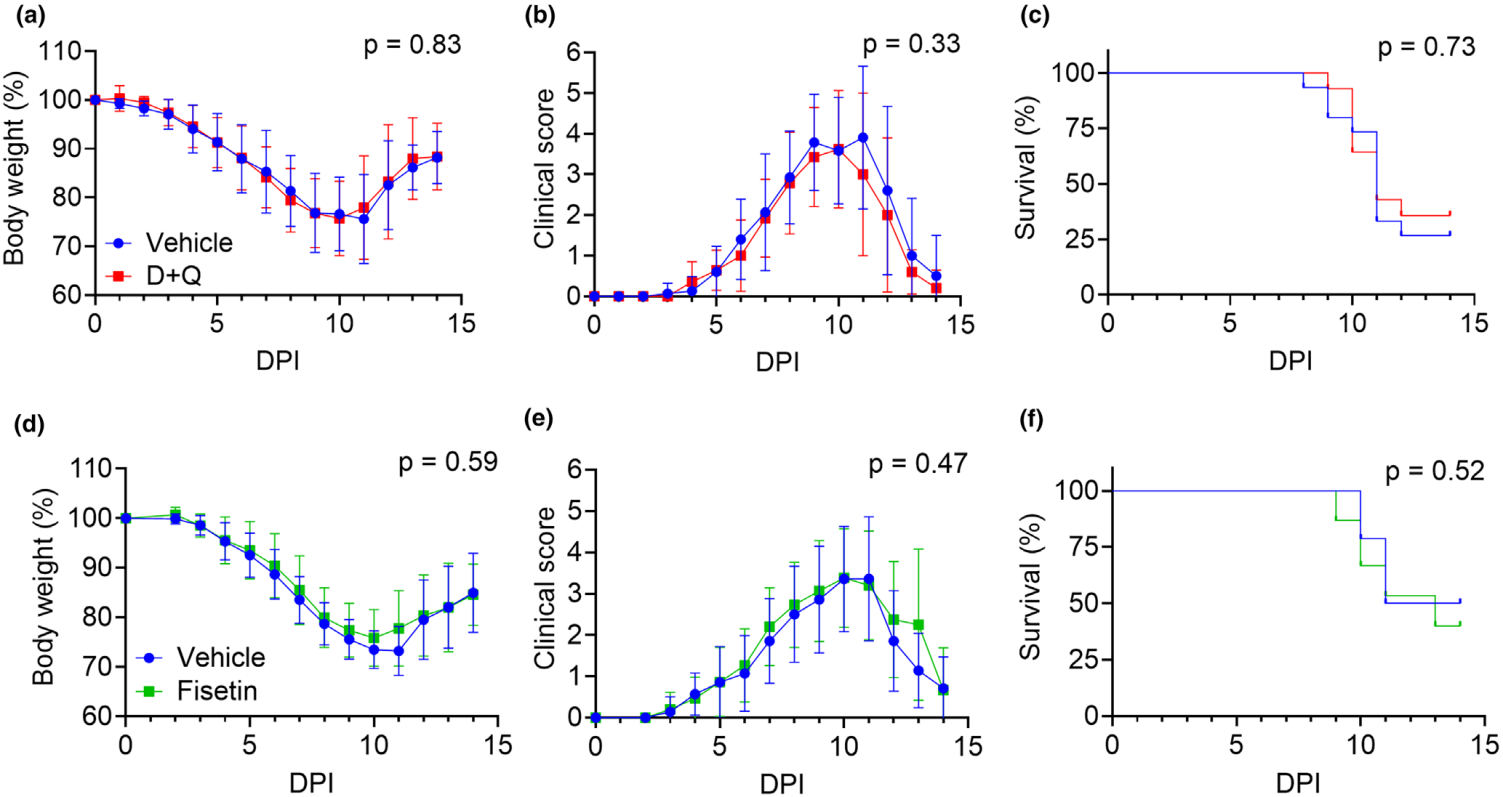
Senolytic pretreatment does not improve morbidity and mortality of IAV infections in aged mice (a–f) Aged female (20–24 months old) mice were pretreated with vehicle, D + Q, or fisetin and infected with IAV (1500 PFU) a week after final oral gavage (*n* = 14–15 per group). Mice health was tracked daily after IAV infection (a, d) Body weight curve of vehicle, D + Q mice or Fisetin. (b, e) Semiquantitative clinical scores. Body weight data and clinical score data were analyzed by Mixed ANOVA. The *p* value represents the effect of treatment compared to vehicle. (c, f) Survival curves comparing Vehicle against senolytic pretreatment of 5 mg/kg dasatanib plus 50 mg/kg quercetin or 100 mg/kg fisetin. Survival curves analyzed by log‐rank test.

Aging has been shown to increase markers of senescence in the lungs, gWAT, and serum of C57BL/6 mice (Biran et al., 2017; Chen et al., 2022; Song et al., 2023). Thus, we sought to determine if the increased number of senescent cells that accumulate with age in the C57BL/6 mice are responsible for worsened outcomes of respiratory infections in aged mice. To investigate the role of senescent cells in respiratory infections in older hosts, we tested if treating mice with senolytics prior to infection (1500 PFU/mouse, or LD_70_) would improve morbidity and mortality in our aged animals (Figure S4a,b). Our results showed that neither D + Q nor fisetin treatment mitigated the morbidity (i.e., weight loss and clinical scores) or mortality of IAV infections in aged mice compared to the vehicle control animals (Figure 4a–f). These results suggest that treatment with senolytics prior to viral infection does not impact the outcomes of influenza infections in aged C57BL/6 mice.

### 2.5 Senolytic treatment during IAV infection does not improve outcomes of aged mice

**FIGURE 5:**
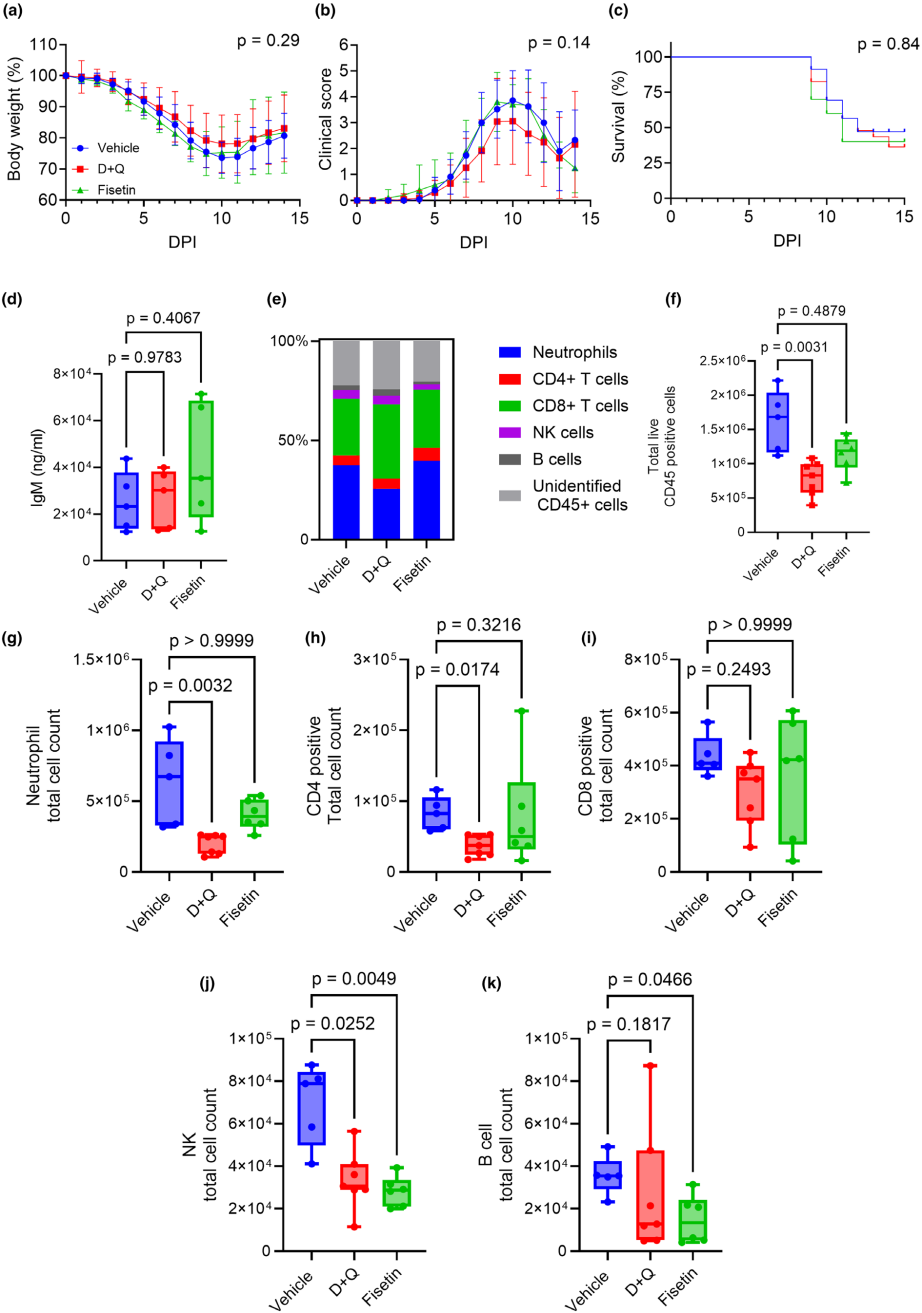
Senolytic treatment during an IAV infection does not improve morbidity and mortality in aged mice. Aged female (18–24 months old) C57BL/6J mice were treated with vehicle (*n* = 23), D + Q (*n* = 23), or Fisetin (*n* = 10) and concurrently infected with 1500 PFU of IAV. (a) Bodyweight curve of treated mice. (b) Clinical scores. Body weight data and clinical score data were analyzed by Mixed ANOVA. The *p* value represents the effect of treatment compared to vehicle. (c) Survival curve data was analyzed by log rank test (d) Quantification of IgM concentration in the lungs (*n* = 5 per group). (e) Immune cell infiltration frequencies at day 8 post IAV infection. (f) Quantification of total live CD45 infiltration in the lung at day 8 post infection in the vehicle and senolytic treated groups (*n* = 5–7 per group). (g–k) Total cell count of neutrophil, CD4+ T cells, CD8+ T cells, natural killer cells, and B cells (*n* = 5–7 per group). Box and whisker plots represent the median and range of the data analyzed by Mann–Whitney test.

Prior publications have shown that coronavirus infections can induce acute senescence which contributes to increased viral load and more severe inflammation during infections (Camell et al., 2021; Lee et al., 2021). Because our results demonstrated that pretreatment with senolytics did not improve outcomes in aged animals, we investigated if senolytic treatment during infection could improve outcomes in aged mice (Figure 5). We infected aged mice with 1500 PFU/mouse (LD_70_) and administered either vehicle or senolytic therapies on day 3, 4, 11, and 12 post‐infection and monitored mice health, mirroring the Camell et al treatment regimen (Figure S4c) (Camell et al., 2021). Similar to our results with pretreatment, neither D + Q nor Fisetin treatment during infection improved morbidity or survival (Figure 5a–c). Although no differences were seen between vehicle, D + Q, and fisetin‐treated groups in survival or morbidity, we examined if senolytic treatments reduced lung damage and/or alter the immune response during infection. To assess lung damage, we quantified IgM concentration in the BALF collected from aged mice infected with 1500 PFU/mouse on day 8. Our results showed no significant difference between vehicle, D + Q (*p* value = 0.9783) and Fisetin (*p* value = 0.4067) in the BALF (Figure 5d). We also measured the frequencies of neutrophils, CD4 T cells, CD8 T cells, natural killer cells, and B cells at 8 days post infection, hypothesizing that the senolytic treatments would increase infiltration of T cells and natural killer cells to levels similar of young mice, but found no significant differences in relative proportions of each cell type (Figure 5e). However, we observed that the total number of live CD45 population was significantly reduced in D + Q treated mice (Experimental vs. control; median ± SD, 829,481 ± 249,971; *p* value = 0.0031) compared to vehicle (1,682,398 ± 455,308) whereas mice treated with Fisetin (1,193,361 ± 253,951; *p* value = 0.4879) did not exhibit significant difference at day 8 post infection (Figure 5f). When each immune cell type was enumerated, we found that D + Q led to significant reduction in numbers of neutrophils (248,636 ± 70,067 vs. 673,553 ± 306,493, *p* value = 0.0032; Figure 5g), CD4 T cells (37,428 ± 14,851 vs. 82,521 ± 23,788, *p* value = 0.0174; Figure 5h), and natural killer cells (30,626 ± 13,681 vs. 78,891 ± 19,232; *p* value = 0.0252; Figure 5j) with no changes in CD8 T cells (350,251 ± 127,672 vs. 408,748 ± 77,166, *p* value = 0.2493; Figure 5i), and B cells (12,815 ± 30,208 vs. 35,456 ± 9169, *p* value = 0.1817; Figure 5k) compared to vehicle. Similarly, Fisetin also led to reduction in some immune cells‐ specifically, a significant decrease in the natural killer cell (28,687 ± 7115 vs. 78,891 ± 19,232, *p* value = 0.0049; Figure 5j) and B cell populations (13,482 ± 11,258 vs. 35,456 ± 9169, *p* value = 0.0466; Figure 5k), with no significant changes in numbers of neutrophils (393,187 ± 106,872 vs. 673,553 ± 306,493, p‐value >0.9999;Figure 5g), CD4 T cells (50,113 ± 77,153 vs. 82,521 ± 23,788, *p* value = 0.3216; Figure 5h), and CD8 T cells (423,041 ± 230,887 vs. 408,748 ± 77,166, *p* value >0.9999; Figure 5i). Thus, our results suggest that neither D + Q nor fisetin improve outcomes of IAV infection in aged mice and might attenuate critical immune cells for antiviral host defense.

Due to the unexpected results, we tested a third senolytic compound, ABT‐263, that has been shown to be effective at improving SARS‐CoV‐2 infections in aged rodent models (Delval et al., 2023). Similar to the previous experiment, we infected aged mice with 1500 PFU/mouse and administered either vehicle or 100 mg/kg of ABT‐263 daily for 6 days starting on day 1 post‐infection and monitored mice health, mirroring the Lee et al treatment regimen (Figure S4d) (Lee et al., 2021). Similar to our results with D + Q and fisetin, ABT‐263 treatment did not improve survival or morbidity (Figure S6a–c). To assess lung damage, we harvested the lung tissues from the mice treated with vehicle or 100 mg/kg ABT‐263 at day 8 post infection. Histopathological analysis was performed on blinded slides, which were evaluated by an accredited pulmonologist, in accordance with American Thoracic Society guidelines (Matute‐Bello et al., 2011). The histological analysis revealed no significant difference in lung damage between vehicle and ABT‐263 treated mice (Figure S6d,e). Altogether, these results suggest that senolytics are unable to improve IAV infection outcomes in aged mice.

### 2.6 Acute senolytic treatment does not reduce senescent cell markers in the lung

We investigated if the inhibitors were biologically active and affecting their known targets or downstream effectors in vitro using normal primary human bronchial/tracheal epithelial (PHBTEN) cells and in vivo using uninfected mice. For the in vitro experiments, we seeded PHBTEN cells and allowed the cells to adhere overnight. We then treated the cells with increasing concentrations of dasatinib (10 nM–100 μM), quercetin (1 μM–200 μM), or fisetin (1 μM–200 μM) independently for ~6 h before harvesting protein for immunoblots. Increasing concentration of dasatinib resulted in a dose‐dependent suppression of p‐Srt1 phosphorylation and p‐AKT, a downstream effector of Src (Figure S5a). Similarly, both quercetin and fisetin decreased p‐Sirt1 expression and suppressed p‐AKT signaling as previously shown by others (Figure S5a) demonstrating that the inhibitors are functional in vitro. To further validate their activity, we treated young mice (2–4 months old) with a dose of 5 mg/kg dasatanib and 50 mg/kg quercetin for 3 days before harvesting the lungs 6 h after the final dose. We homogenized the lungs and measured p‐AKT activity within the lung lysate by immunoblot. In support of the primary cell line data, the in vivo mouse experiment shows a reduction of p‐AKT detection (*p* value = 0.0009; Figure S5b,c). Together this data validates that the senolytic agents suppress their molecular targets and are metabolically active in vivo in the lungs of treated mice.

**FIGURE 6:**
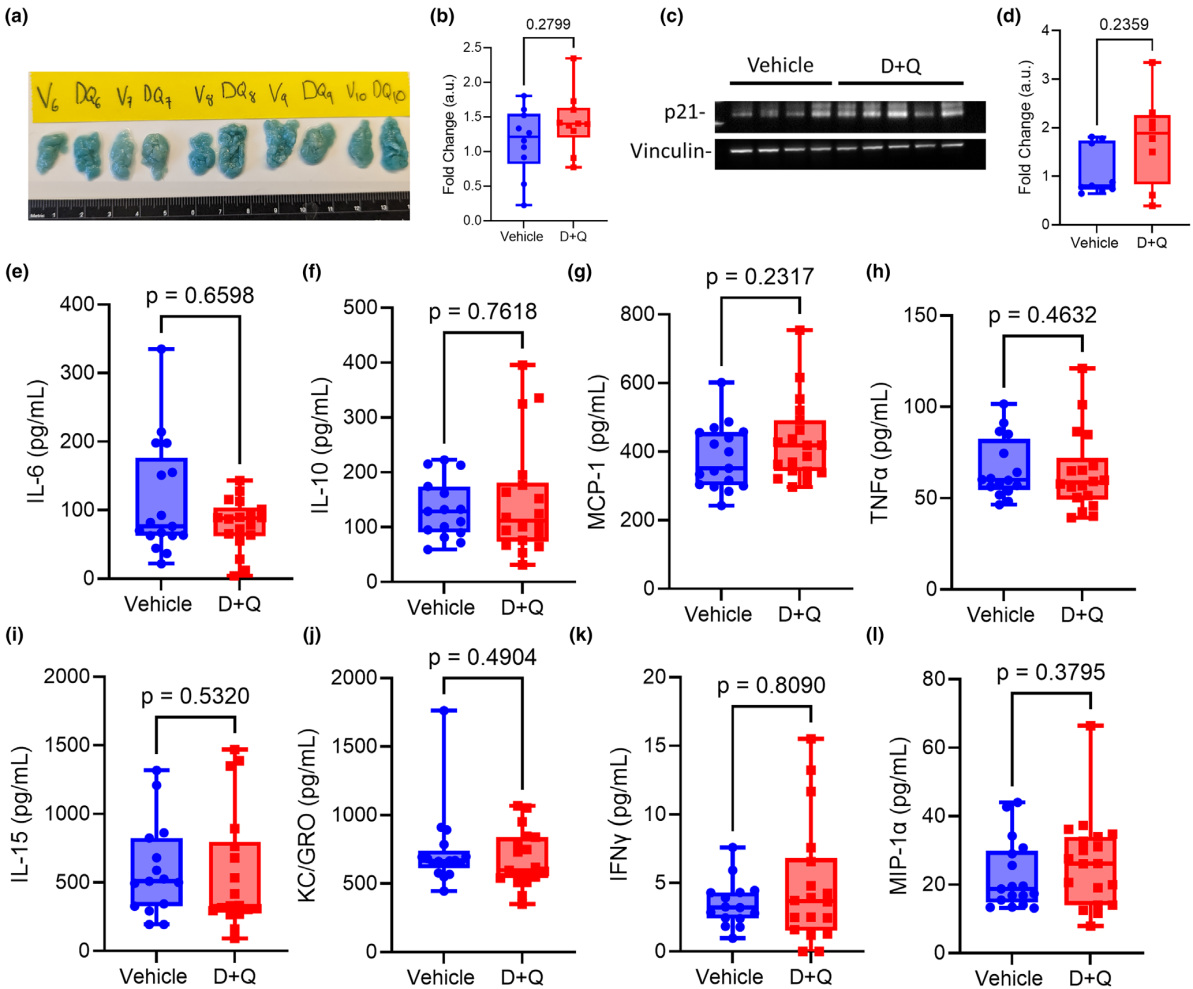
Dasatanib and quercetin is active in the lung of mice after oral gavage but is not reducing senescence markers. Aged female (19 months old) mice were given the full pretreatment D + Q regiment. Three to five days after the last dose gWAT, lungs and sera were collected and processed for whole mount β gal staining, immunoblots or MSD multi‐spot assay. (a) Gonadal white adipose tissue stained with X‐Gal to detect b‐galactosidase activity (*n* = 10 mice per group). (b) Densitometry analysis was performed on stained adipose tissues using Fiji (*n* = 10 mice per group). (c) Total Lung lysates (25 μg/lane) were analyzed by SDS‐PAGE and immunoblot. (d) Densitometry analysis of lung homogenate immunoblot (*n* = 8–9 per group). (e–l) Serum samples from young and aged mice (*n* = 18–19 per group) were used on the MSD multi‐spot assay to detect SASP factors. Box and whisker plots represent the median and range of the data were analyzed by Mann–Whitney test.

We next tested if the inhibitors reduced senescence markers in vivo. We treated aged mice as in Figure S4a,d but harvested the gWAT, the lungs and serum 3–7 days after the final dose of D + Q, ABT‐263 or vehicle. Using the same methods for the studies in Figure 1 to detect the differences between our young and aged mice groups, we first determined whether D + Q or ABT‐263 reduced β‐gal staining. Visually, no difference was seen between the vehicle and senolytic treated gWAT samples collected (Figure 6a and Figure S7a) and quantification detected no difference between the two groups (D + Q *p* value = 0.2799; ABT‐263 *p* value = 0.1905; Figure 6B and Figure S7b). Additionally, we determined if D + Q or ABT‐263 treatment reduced expression of p21 in the lung tissue lysate. Concordant with the gWAT data, we found that the senolytic treatment did not reduce the expression of p21 (D + Q vs. Vehicle *p* value = 0.2359; ABT‐263 vs. Vehicle *p* value = 0.9890; Figure 6c,d and Figure S7c,d). Finally, we measured nine SASP factors from Figure 1 by MSD multi‐spot assay, including the three we previously found to be differentially expressed between our young and aged mice groups. We found that D + Q and ABT‐263 had no effect in reducing the levels of the SASP factors (Figure 6e–l and Figure S7e–l). Collectively, our data indicates the compounds are exerting their anticipated effects on their targeting signaling pathways but are not reducing markers of senescence in vivo.

## 3 DISCUSSION

Several studies have shown that aged mice have worse outcomes of influenza, respiratory syncytial virus, and SARS‐CoV‐2 infections, mirroring human data (Camell et al., 2021; Kulkarni et al., 2019; Wong et al., 2014). The immunopathology underlying severe respiratory infections is thought to be caused by the increased inflammatory response of the innate immune system and the functional impairment of the adaptive system (Pietrobon et al., 2020). In previous work, we associated poor IAV outcomes in aged mice with more senescent alveolar epithelial cells and higher levels of neutrophil attracting chemokines CXCL1 and CXCL2 (Kulkarni et al., 2019). In this study, we found fewer leukocyte numbers, an imbalance of neutrophils and CD8+ T cells and increase mortality and lung injury in aged mice despite no difference in viral load. We therefore aimed to test the contribution of senescent cells to outcomes and immune dysregulation during influenza infections. We hypothesized that senolytic treatment would reduce neutrophil recruitment, decrease injury, and improve outcomes but our findings showed no improvement in morbidity or mortality with senolytic treatment in aged mice.

Our study aimed to build on previous research that suggested senolytics improve outcomes in SARS‐CoV‐2 infections in rodent models ranging from 2 to 24 months (Camell et al., 2021; Delval et al., 2023; Lee et al., 2021; Pastor‐Fernandez et al., 2023). These models showed that removing senescent cells reduces inflammation and improves survival outcomes by mitigating the hyper‐inflammatory response associated with cellular senescence (Camell et al., 2021; Delval et al., 2023; Pastor‐Fernandez et al., 2023). The hyper‐inflammatory response is characterized by increased levels of inflammatory molecules like IL‐6 and MCP‐1, reduced infiltration of T cells and B cells into lungs and impaired lymphocyte function during infection. Our study supported the concept that aged mice had more severe outcomes as a result of cellular senescence. Thus, we tested if the removal of senescent cells before infection or during infection would improve outcomes in our aged C57BL/6 IAV infection model. To our surprise, we showed no difference in weight loss, clinical score, lung injury, or survival with senolytic treatments.

Our study emphasizes the need to critically and rigorously appraise the potential of senolytics as therapeutic options for various respiratory infections and other diseases. We tested three established treatments: D + Q, fisetin, and ABT‐263. Furthermore, we treated aged mice with two different regimens of D + Q and fisetin (i.e., pretreatment and during infection). Surprisingly, we did not find evidence of improved outcomes of influenza infection with any of these therapeutic approaches in our aged mice. Interestingly, we note that senolytic treatment did not reduce markers of senescence in aged mice as measured by β‐gal staining on gWAT, p21 protein expression in the lungs nor SASP factors in the sera of senolytic treated mice compared with vehicle treated controls. However, the biologic activity of dasatanib, quercetin, and fisetin inhibitors was confirmed through the suppression of the inhibitors' known targets and reduced infiltration of various immune cells in the lung during infection of treated mice. Overall, our data suggest that D + Q and fisetin were ineffective at decreasing senescence despite having bioactivity in vitro and in vivo. Thus, the potential of senolytics to improve outcomes of respiratory infections will likely need further mechanistic investigation and rigorous assessment of pre‐ and post‐treatment senescence markers.

We considered three potential reasons as to why we did not see alterations in outcomes. The first potential reason is that the dosage and duration of treatment were not sufficient in our infection model. Our data showed that our dosing regimen of D + Q and ABT‐263 was insufficient to cause a reduction of the senescence markers measured. However, we used regimens and inhibitor concentrations that have been previously published in literature with positive results in the SARS‐CoV‐2 models (Camell et al., 2021; Lee et al., 2021). Further investigation is needed particularly since the treatment regimens of senolytics vary significantly in duration and concentrations in the literature, and direct evidence that senolytics exert their salient effects via reducing the burden of senescence are not consistently provided (Camell et al., 2021; Lee et al., 2021; Pastor‐Fernandez et al., 2023). A second potential explanation is that there could be a specific time point during IAV infection where the treatment is effective at improving outcomes in aged mice. This hypothesis is supported by our previous data which demonstrated that late (Day 6 post infection) neutrophil depletion timing is crucial for improved outcomes in aged mice during influenza infections while early (Day 0 post infection) neutrophil depletion had no effect on outcomes (Kulkarni et al., 2019). However, our senolytic treatments not only show decreased total neutrophil infiltration on day 8 post‐infection after D + Q treatment but also reduce infiltration of other innate and adaptive immune cells potentially counteracting the benefit of the neutrophil depletion in the late stage of infection. A third possible explanation is that the positive outcomes of senolytic treatment during SARS‐CoV‐2 are pathogen‐specific. As an example, both Camell et al. and Delval et al. demonstrated that the senolytic treatment reduces the expression of ACE2 expression, the entry receptor of SARS‐CoV‐2. This reduction in ACE2 expression could potentially explain the lower viral titters during infection and improved outcomes seen with senolytic treatment (Camell et al., 2021; Delval et al., 2023), even in young animals. Unlike SARS‐CoV‐2, ACE2 does not play a role in the replication of IAV. Thus, if the improved outcomes of SARS‐CoV‐2 are dependent on the reduced expression of ACE2, we would not expect improved outcomes in IAV infections following senolytic treatment. However, the genetic removal of senescence using the INK‐ATTAC transgenic mouse model and the improved outcomes in a β‐coronavirus mouse infection model provide evidence that senolytics can still have benefit independent of ACE‐2 mediated mechanisms (Camell et al., 2021). Nonetheless, it is important to note that senolytics can exert direct antiviral effects that are independent of their role in targeting cellular senescence, and therefore may not have the substantive benefit in other viral infections as was observed in SARS‐CoV‐2 (Chaves et al., 2022; Jiao et al., 2023; Roy et al., 2024).

In support of our findings that D + Q does not improve IAV infection outcomes in an aged model, Torrance et al. demonstrated that treating mice with D + Q before IAV infection did not improve morbidity nor reduce lung damage in aged mice (Torrance et al., 2023). Additionally, Torrance et al. also demonstrated that D + Q treatment resulted in reduced IAV‐specific CD8 cells and no alteration in the T‐cell memory response at day 30 post‐infection (Torrance et al., 2023). Our data, although not measuring IAV‐specific CD8, also demonstrated that D + Q treatment led to a nonsignificant reduction in the infiltrating CD8 T cells. Our experiments exploring the differences in immune infiltration between our young and age mice groups demonstrated that aging results in increased neutrophil infiltration and reduced CD4 T cell, CD8 T cell, and NK cell infiltration around the peak of infection. Thus, we would have expected that senolytic treatments to reverse the aging phenotype and promote an increase infiltration of CD4, CD8, and NK cells. However, our results demonstrate that the senolytic treatments did not increase infiltration of these cells but exaggerated the suppression that occurs with aging, making it unlikely that acute senolytic treatments would have positive benefits for IAV infections. Notably, the reduction of immune infiltration into the alveolar space following senolytic treatments appeared to be independent of direct senolysis. This effect may be attributed to direct interaction of the inhibitors with either immune cells or tissue resident cells that could affect cell recruitment or migration.

A recent publication from Chang et al., demonstrated that ABT‐263 could rejuvenate aged hematopoietic stem cells in mice by clearing senescent cells and improving immune function. The difference between our findings and those of Chang et al. may stem from variations in the duration of treatment and timing at which the effects of senolytics were assessed (Chang et al., 2016). In contrast to Chang et al., who focused on prolong senolytic exposure, our study applied senolytics post infection acutely, a clinically relevant approach for elderly patients facing acute viral infections. Our data suggest that acute senolytic treatment does not immediately restore immune function; instead, it may suppress immune cell infiltration, reducing CD4 T cells, CD8 T cells, and natural killer cell presence in the alveolar space during infection, all of which are essential for effective viral clearance (Bender et al., 1992; Gazit et al., 2006; Mozdzanowska et al., 2000; Stein‐Streilein & Guffee, 1986). This distinction raises the question whether long‐term senolytic treatment could have broader immune altering effects.

Consistent with other studies, we found that the aged mice relative to young mice had a significant reduction of total infiltrating leukocytes caused by a contraction of the number of CD4 T cells, CD8 T cells, and natural killer cells number in the alveolar space (Figure 3b–h). Interestingly, we did not find increased absolute numbers of neutrophils compared to young mice, as we previously reported during IAV infection (Kulkarni et al., 2019). We believe the lack of neutrophil induction is due to our examination of the lung infiltrates profile at day 8 post infection rather than an earlier timepoint where the neutrophils would be infiltrating the alveolar airspace. The reduced number of CD4 T cells and CD8 T cells are likely as the result of thymic involution that occurs with aging that causes reduced number of naïve lymphocytes in the blood. The sum of the reduction of T cells and natural killer cells with aging likely contributed to the higher mortality in our aged mice as each of these cells have been demonstrated to play a vital role in controlling IAV infection. Further observations are needed to determine if the functionality of the noted immune cells was altered.

The role of cellular senescence in shaping immune responses to respiratory infections is a complex and rapidly evolving field. Emerging evidence suggests that senescent cells can both promote and impair immune function, depending on the context. Recent studies, including our current study, demonstrate that senolytic treatment with dasatinib and quercetin did not enhance immune responses to influenza in aged mice, challenging the assumption that removing senescent cells universally improves immune function (Torrance et al., 2023). In contrast, recent findings suggest that the genetic clearance of senescent cells using the p16‐3MR mouse model enhanced viral clearance but also significantly impaired the formation of immune memory following influenza infection (Torrance et al., 2024). These results indicate that eliminating senescent cells may have unintended consequences, potentially disrupting other processes critical for immune protection. Additionally, research from the Haynes laboratory has shown that senolytic treatment can reverse age‐related, senescence‐induced changes in CD4+ T cells, suggesting a potential mechanism by which senolytics could improve outcomes (Lorenzo et al., 2022). Collectively, these findings underscore the need for a nuanced understanding of senescent cell biology in the context of specific immune cell types during respiratory infections. The findings that different senescent cell types may exert distinct and even opposing effects on immunity suggest that a one‐size‐fits‐all approach to senolytic therapy may be inadequate as in some contexts, senescent cells may contribute to an immune‐suppressive milieu that hinders pathogen clearance, while in others they may play a protective role in limiting excessive inflammation.

A few limitations of our study warrant consideration. First, although we did not observe a reduction in cellular senescence markers following senolytic treatment, these findings may be due to the relatively young and healthy status of our aged mice, suggesting that age related senescence under healthy conditions may be less pronounced. A model incorporating additional comorbidities (e.g., obesity, cigarette smoke exposure, etc.) could yield different results, better reflecting the senescent profiles typically seen in clinically relevant aging conditions. Additionally, as our study exclusively utilized female mice, it may not capture sex‐specific differences in response to senolytic treatments. A third limitation is that we did not evaluate the long‐term effects of senolysis on infection sequelae, such as memory immune responses or lung injury recovery, an important area for future investigations. Our protocol employed a lethal viral dose (70%–80% mortality in aged mice) and used a heterogeneous age range of aged animals, which may have masked potential benefits of senolytic therapy, Future studies using additional sublethal doses in aged animals could reveal more positive effects of senolytics on IAV infection outcomes. Likewise, a narrower age range among aged study animals could reduce biological variability, improving sensitivity to detect subtle differences.

To summarize, here we report that senolytic treatments are not effective at improving outcomes of IAV infections in aged animals. Furthermore, our results suggest that the acute treatments of senolytics in aged mice are not able to effectively reduce markers of senescence. Our results suggest that acute senolytic treatments are unable to revert the effects of aging in the immune system during influenza infections. Further investigation is needed to understand the long‐term effects of senolytics on the outcomes of other infectious pathogens and the mechanisms underlying the effectiveness of senolytic therapies in a pathogen‐specific manner.

## 4 METHODS AND MATERIALS

### 4.1 Animal handling and influenza infection

All mice used in this study where female as male mice are resistant to IAV infections. Female young (2–4 months) and aged (18–24 months) C57BL/6 mice were either purchased from Charles River Laboratories or received from the National Institute on Aging (NIA). Upon arrival, the mice were monitored for signs of illness for 1 week, and any sick animals were removed from the study. Mice were housed at the North Campus Research Complex at the University of Michigan in a specific pathogen‐free facility with a 12‐h light–dark cycle. The animal procedures were approved by the Animal Care and Use Committee (PRO00010704). All mice were fed food and water ad libitum. In the senolytic experiments, mice were age‐matched but otherwise randomly distributed into groups. Mouse IAV infections were carried out in a BSL2 containment facility at the University of Michigan. The mice were anesthetized with isoflurane and intranasally infected with 500 PFU or 1500 PFU of IAV in a 40 μL inoculum volume. Following infection, the mice's weight and clinical scores were monitored daily. Clinical score was determined as previously described (Chen et al., 2022). Briefly, neurological and general symptoms are scored from 0 to 5 based on mice's behavior including ruffle, hunch, stiff gate, tremor, mobilities, and convulsions. Euthanasia was performed if the mouse lost more than 30% of its original weight. The senolytic compounds were dissolved in a mixture of 30% PEG‐400, 60% Phosal‐50, and 10% ethanol. Dosing concentrations (in mg/kg/d) were administered in 100 μL volumes, with specific schedules illustrated in Figure S4a–d, as they vary by experiment.

### 4.2 Primary cell culture

The primary bronchial/tracheal epithelial cells were purchased from ATCC (PCS‐300‐010). The cells were derived from a white 18‐year‐old nonsmoker male. The primary epithelial lung cells were maintained in Airway Epithelial Cell Basal Medium (PCS‐300‐030) with Bronchial Epithelial Cell Growth Kit (PCS‐300‐040) as recommended by the manufacturer without antibiotics.

### 4.3 Wholemount X‐gal staining

Freshly isolated gonadal white adipose tissue was stained using the Cell signaling X‐gal staining kit (CST #9860). Soon after harvest, the tissue was harvested and placed in fixative solution for 30 min at room temperature. The tissue was then rinsed with PBS and washed with tissue detergent buffer (0.02% Igepal (Millipore #188696), 0.01% Sodium Deoxycholate, and 2 mM MgCl_2_ (Invitrogen #AM95300G) in 0.1 M phosphate buffer). The adipose tissue was stained by immersion in 1 mg/mL X‐gal staining solution overnight. After staining tissue was fixed in 4% PFA in PBS at 4C. Tissues were imaged and analyzed with Fiji software.

### 4.4 Protein isolation and immunoblotting

Lung and gonadal white adipose tissue samples were lysed on ice in TPER buffer (Thermo Scientific #78516) while cell culture samples were lysed with RIPA buffer (Thermo Scientific #89900) supplemented with 1% protease inhibitor (Sigma‐Aldrich P8340) and 1% phosphatase inhibitor (Sigma‐Aldrich P5726) using the tissue‐lyser III. Samples were centrifuged at 12 K × g for 15 min at 4C and supernatants were transferred to fresh tubes. Protein concentrations were determined using pierce BCA assay (Thermo Scientific #23227). Sample loading buffer was added to samples. Total proteins were subjected to 4%–12% sulfate‐polyacrylamide gel electrophoresis. Protein was transferred to PVDF membrane using the semidry transfer system (Invitrogen Iblot 2). Membranes were blocked with 5% bovine serum albumin in Phosphate buffered saline with 0.1% tween‐20. Primary antibodies were incubated with membrane overnight at 4°C. HRP conjugated secondaries were incubated for 1 h at room temperature. Antibodies purchased from Cell Signaling: p‐SIRT1 (#2314S), SIRT1 (#9475S), p‐AKT (#4060S), pan AKT (#4691S), and GAPDH (#2118L) were used according to the manufacturer's recommendations. The p21 (#6246) antibody was purchased from Santa Cruz Biotechnologies. HRP‐conjugated anti‐rabbit secondary antibodies (Cell Signaling #7074S). Immunoblots were imaged on Bio‐Rad ChemiDoc and analyzed with Fiji.

### 4.5 MSD multi‐spot SASP assay

MSD UPLEX MULTI‐SPOT Assay (#K15067M‐Z) was run according to manufacturer's recommendations. Briefly, to prepare plates for ELISA, multiplex coating solution was prepared by combining each biotinylated antibody with its respective linker, followed by a 30‐min incubation at room temperature. Subsequent to this, the stop solution was added. 50 μL of this multiplex coating solution was dispensed into each well and sealed before undergoing overnight incubation at 4°C with gentle agitation. Sample or calibrator standards were diluted using Diluent 41 and added to each well. Plates were then sealed and incubated at room temperature with agitation for an hour. After three wash cycles with 1X MSD wash buffer, the detection antibody solution was introduced to each well, sealed, and subjected to a further hour of incubation with shaking set to 700 rpm. Another three washes preceded the addition of MSD Gold Read Buffer B to each well, after which the plate was promptly analyzed on an MSD instrument.

### 4.6 Viral RNA measurements

Viral RNA in the mouse lungs was measured using a TaqMan RNA assay. First, RNA was isolated by homogenizing frozen lung tissue with a mechanical homogenizer for 10–20 s. The homogenized lung samples were then centrifuged at approximately 10,000 × *g* for 10 min at 4°C. RNA was extracted from 100 μL of the lung homogenate using a Qiagen RNeasy kit (Qiagen #74104), following the manufacturer's instructions. Next, cDNA was synthesized from 2 μg of RNA using a High‐Capacity cDNA synthesis kit (Fisher# 43‐688‐14). Each mRNA sample was diluted 2‐fold and used for the TaqMan qPCR reaction. The pIRS426‐M2M1‐10X HIS plasmid was used to create the standard curve for quantifying the number of viral genomes in the qPCR reaction.

### 4.7 Mouse IgM Elisa

The IgM level of mouse BAL was measured using IgM Mouse ELISA Kit (Invitrogen 88‐50470). High‐Affinity protein binding plates were coated with the capture antibody and blocked following the manufacturer's instructions. Samples, standards, and Assay Buffer A were then added to the respective wells, and the plates were sealed and incubated at room temperature for 2 h with shaking at 400 rpm. Post‐incubation, the detection antibody was added and incubated for another round. Plates were read at 450 nm after the addition of Substrate solution and Stop solution.

### 4.8 Flow cytometry

Mice were euthanized and the trachea was intubated with a 23‐gauge polyethylene tubing attached to a syringe. Lungs were inflated 5 consecutive times with 1 mL volumes of 5 mM EDTA PBS solution and lavage fluid was withdrawn from the lung and combined to a single tube. Cells were pelleted at 500 g for 5 min and retained for flow cytometry analysis. Cells pellets were resuspended in LIVE/DEAD Fixable Aqua stain and incubated with anti‐CD16/32 to block Fc receptors. Cells were stained with antibodies targeting extracellular proteins to identify immune cell infiltrates in the alveolar air space. The flow cytometry antibodies were purchased from BioLegend: CD3 BV650 (#100229), F4/80 APC (#157306), CD8 PE/Cyanine 7 (#155018), Ly6G BV421 (#127628), CD4 APC/Cyanine 7 (#100414), CD19 PerCP/Cyanine 5.5 (#152406), CD45 Alexa Fluorophore 488 (#103127), and CD49b PE (#108908). After, cells were washed and fixed in 2% PFA. Samples were acquired on a BD FACSymphony A5 and data analyzed using FlowJo v10.8.1. To determine total cell count, a defined volume of antibody‐stained single cell suspension was collected by the flow cytometer and the counts were adjusted based on the total volume of fluid recovered from BAL samples. The gating strategy is shown in supplemental figures (Figure S2).

### 4.9 Lung histology analysis

Whole lung H&E‐stained were examined for histologic scoring based on the guidelines provided by the American Thoracic Society workshop report for the measurement of experimental lung injury in animals (Matute‐Bello et al., 2011). Briefly, the reviewer was blinded to the sample identities, Due to the heterogeneous distribution of lung injury, at least 10 sections were randomly selected from each sample for analysis, ensuring that at least two sections were obtained from each lobe of the lung. For each section, approximately 5 high‐power fields (with >50% alveolar spaces) were randomly analyzed at 100× magnification. A score from 0 to 2 (with 2 being the most severe) was assigned for the following criteria: (A) neutrophils in the airspace, (B) neutrophils in the interstitium, (C) presence of hyaline membranes, (D) proteinaceous debris in the airspace, and an overall injury score for each sample was calculated using the formula: Score = [(20 × A) + (14 × B) + (7 × C) + (7 × D) + (2 × E)]/(number of fields × 100). The overall scores range from 0 to 1, with a score of 1 indicating more severe lung injury.

### 4.10 Statistics

Statistical analyses were performed using GraphPad Prism version 10.8.1. To compare two groups (e.g., young vs. aged mice), we used the Mann–Whitney nonparametric test. For body weight and clinical scores, a mixed effect model was employed to assess significance between groups/treatments. To compare multiple groups in box and whisker plots, we conducted a one‐way Brown‐Forsythe ANOVA followed by Dunnett's T3 multiple comparisons test. Survival data were analyzed using the log‐rank test. p Values are indicated in the figures. Illustrations were created using BioRender.

## AUTHOR CONTRIBUTIONS

A.J.L., K.W., M.S., D.G., and J.D. designed research; A.J.L., K.C., K.W., M.W., N.P., and X.Z. performed research; A.J.L., J.D., and K.W. analyzed data; and A.J.L., K.C., M.B., B.B.M., M.S., D.G., and J.D. wrote the paper. All authors approved the final manuscript.

## FUNDING INFORMATION

This study was supported by the US National Institutes of Health awards Al128347 (D.R.G. and J.C.D.), AG028082 (D.R.G.), HL155169 (D.R.G)., R35HL144481 (B.B.M), T32HL007749 (A.J.L), and VA ORD I01BX004565 and I01BX005447 (to J.C.D).

## CONFLICT OF INTEREST STATEMENT

There are no conflicts of interest.

## Supporting information


Figures S1–S7.

## Data Availability

The data that support the findings of this study are available from the corresponding author upon reasonable request.
