# Supplementary material for: Senolytic treatment attenuates immune cell infiltration without improving IAV outcomes in aged mice
Source: Aging Cell. 2025 Jan 3;24(4):e14437. doi: 10.1111/acel.14437 (PMC11984683; doi:10.1111/acel.14437)
Supplement: Supplementary file 1 — Figures S1–S7. [file ACEL-24-e14437-s001.zip › Supplemental Figure legend_resubmission 2 Final_011524.docx]

**Supplemental Figure 1. Viral Load in the lungs of infected mice with and without senolytics.** Young (2-4 months) and aged (20-21 months old) female mice were infected with 500 PFU/mouse or 1500 PFU/mouse, and lung samples were collected on days 8 and 16 post-infection. RNA was extracted from the lungs, and viral RNA levels were quantified using qPCR with a standard curve. (A) Viral load comparison in young mice (n = 5 per time point) at day 8 and day 16. (B) Comparison in aged mice (n = 5 per time point) at day 8 and day 16. (C) Joint comparison of viral loads between young and aged mice at both day 8 and day 16. Additionally, viral loads in aged mice treated with vehicle (n = 5–6 per time point), D+Q (n = 6 per time point), or fisetin (n = 6 per time point) were compared at days 8 (C) and 16 (D).

**Supplemental Figure 2. Flow cytometry cell differential of gating strategy.** Bronchoalveolar lavages were performed to collect infiltrating cells from the lungs during infection. Cells were stained with live/dead Aqua dye, followed by a panel of antibodies, including anti-CD45-Alexa Fluor 488, anti-CD19-PerCP/Cy5.5, anti-F4/80-APC, anti-CD4-APC/Cy7, anti-Ly6G-Brilliant Violet 421, anti-CD3-Brilliant Violet 650, anti-CD49b-PE, and anti-CD8-PE-Cy7. Our gating strategy began with selecting CD45+, followed by cell gating using SSC-A and FSC-A. All subsequent subpopulation percentages were calculated as a percentage of live CD45+ single cells. Live CD45+ single cells were gated for Ly6G, which were then split into Ly6G+ (neutrophils) and Ly6G- cells. The Ly6G- cells were further gated into CD4+CD8- (CD4 T cells) and CD4-CD8+ (CD8 T cells). The Ly6G-CD4-CD8- cells were then gated for CD49b to identify Ly6G-CD4-CD8-CD49b+ (NK cells). Finally, Ly6G-CD4-CD8-CD49b- cells were divided into CD19+ (B cells) and CD19- (unidentified CD45+ cells).

**Supplemental Figure 3. Senescence markers increase with aging.** Young (2-4 months old) and aged (20-21 months old) mice were infected with a sublethal dose of IAV (500 PFU/mouse) and BALF was harvested to measure immune infiltrates into the lung airspace during an IAV infection. BALF cell infiltrate frequencies at (A) day 8 post infection (n = 5 per group) and (B) day 16 post infection (n = 5 per group) as measured using flow cytometry. (C-H) Total cell count of neutrophil, CD4+ T cells, CD8+ T cells, natural killer cells and B cells on day 16 post infection (n = 5 per group). Box and whisker plots represent the median and range of the data, which were analyzed by Mann-Whitney test.

**Supplemental Figure 4. Senolytic treatment regiments.** (A) Depiction of 5 mg/kg dasatanib plus 50 mg quercetin pre-treatment dosing schedule (B) Depiction of 100 mg/kg fisetin pre-treatment dosing schedule. (C) Depiction of 5 mg/kg dasatanib plus 50 mg Quercetin or 100 mg/kg Fisetin dosing schedule during IAV infection.

**Supplemental Figure 5. Dasatinib and Quercetin are bioactive in vitro and in vivo**. (A) Primary normal human bronchial/tracheal epithelial cells were seeded at a low density (~2.7x10^4 cells/cm^2) and treated with increasing concentrations of dasatanib, quercetin or fisetin for 6 hours before protein lysates were harvested and subjected to SDS-PAGE and IB. (B) Young mice (2-4 months old) were given three doses of 5 mg/kg dasatanib + 50 mg/kg quercetin before the lungs were harvested 6 hours after final dose. Total Lung lysates (25 ug/lane) of mice treated with vehicle, D+Q were analyzed by SDS-PAGE and immunoblot. (C) Densitometry quantification of lung lysate using Fiji (n = 4-5 per group). Data was analyzed by Mann-Whitney test.

**Supplemental Figure 6. ABT-263 treatment during an IAV infection does not improve morbidity and mortality in aged mice*.*** Aged female C57BL/6 mice (18-19 months old) were treated with vehicle (n = 8) or ABT-263 (n = 10) and concurrently infected with 1500 PFU of IAV. (A) Bodyweight curve of treated mice. (B) Clinical scores. Body weight data and clinical score data were analyzed by Mixed ANOVA. The p-value represents the effect of treatment compared to vehicle. (C) Survival curve data was analyzed by log rank test. (D) Histological scoring of lung damage (n = 3 per group) (E) Representative images of lungs from mice treated with vehicle or ABT-263 collected at day 9 post infection.

**Supplemental Figure 7.** Aged female C57BL/6 mice (18-19 months old) were given ABT-263 daily for 6 consecutive days. A week after the last dose gWAT, lungs and sera were collected and processed for whole mount β gal staining, immunoblots or MSD multi-spot assay. (A) Gonadal white adipose tissue stained with X-Gal to detect b-galactosidase activity (n = 4-5 mice per group). (B) Densitometry analysis was performed on stained adipose tissues using Fiji. (C) Total Lung lysates (25 ug/lane) were analyzed by SDS-PAGE and immunoblot. (D) Densitometry analysis of lung homogenate immunoblot (n = 5 per group). (E-L) Serum samples from young and aged mice (n = 3-5 per group) were used on the MSD multi-spot assay to detect SASP factors. Box and whisker plots represent the median and range of the data, which were analyzed by Mann-Whitney test.
