# Supplementary figures and images for: Senolytic treatment attenuates immune cell infiltration without improving IAV outcomes in aged mice
Source: Aging Cell. 2025 Jan 3;24(4):e14437. doi: 10.1111/acel.14437 (PMC11984683; doi:10.1111/acel.14437)

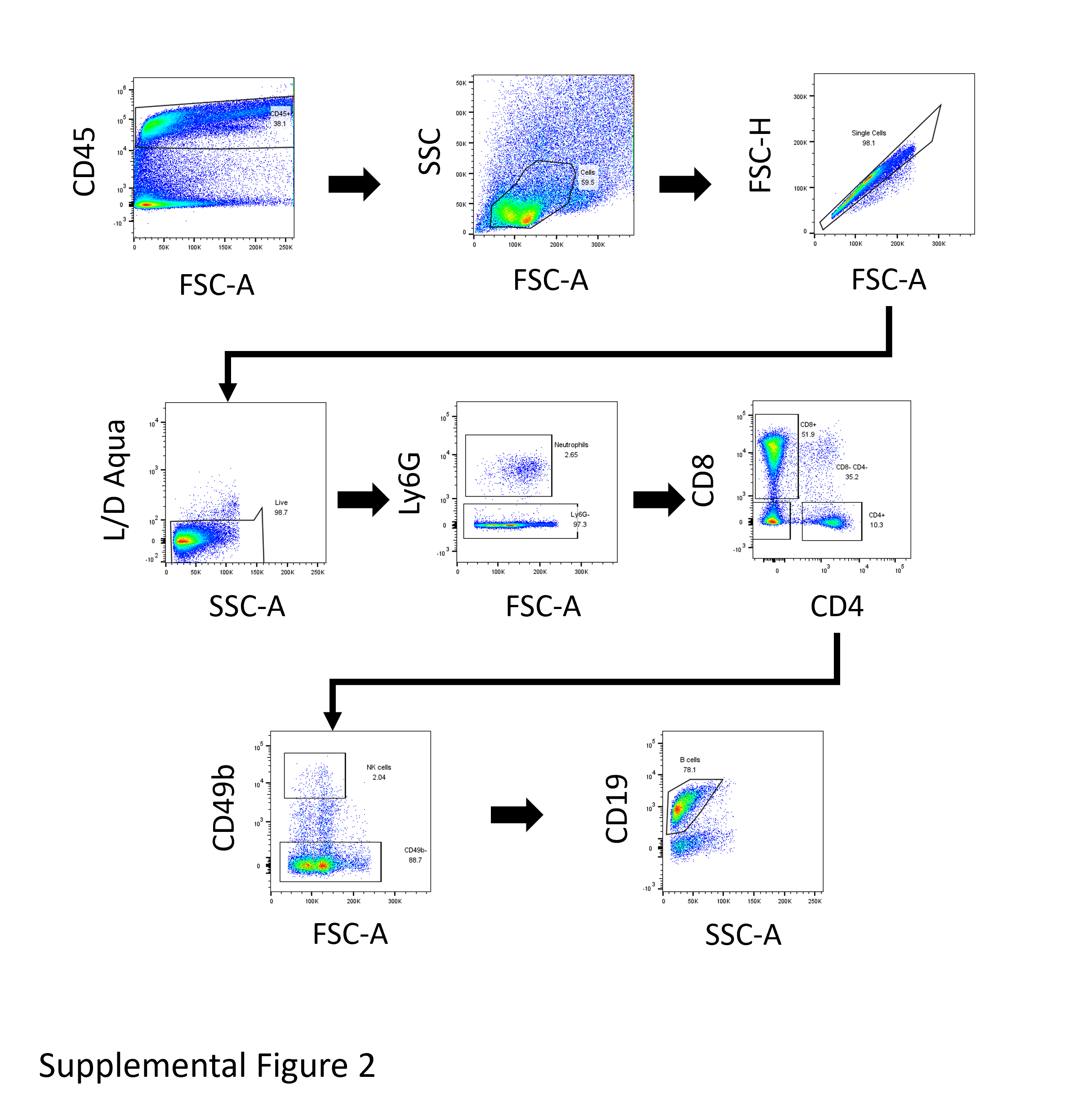

Supplement: Supplementary file 1 — Figures S1–S7. [file ACEL-24-e14437-s001.zip › Supplemental figure 2_resubmission.tif]

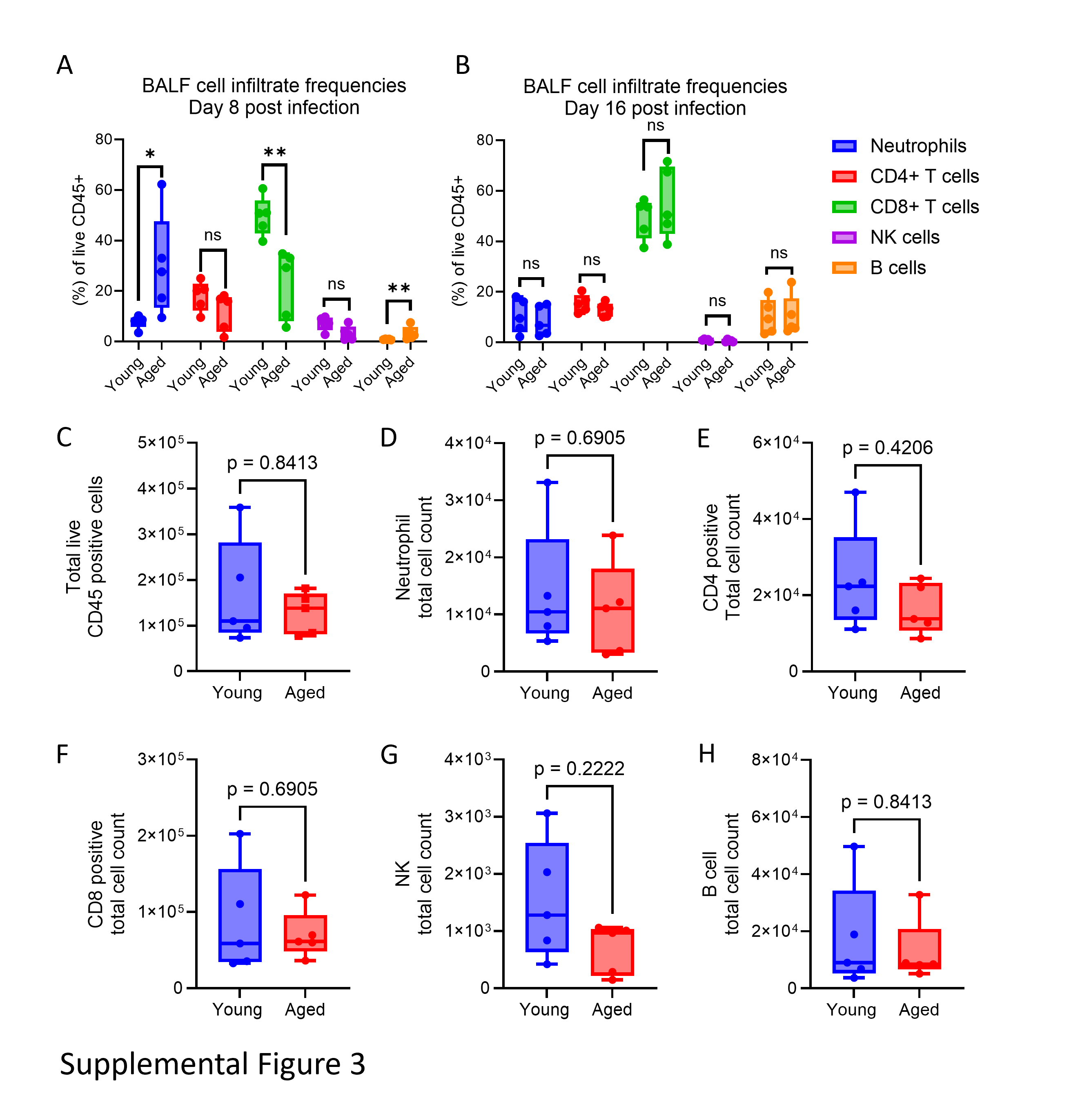

Supplement: Supplementary file 1 — Figures S1–S7. [file ACEL-24-e14437-s001.zip › Supplemental figure 3_resubmission.tif]

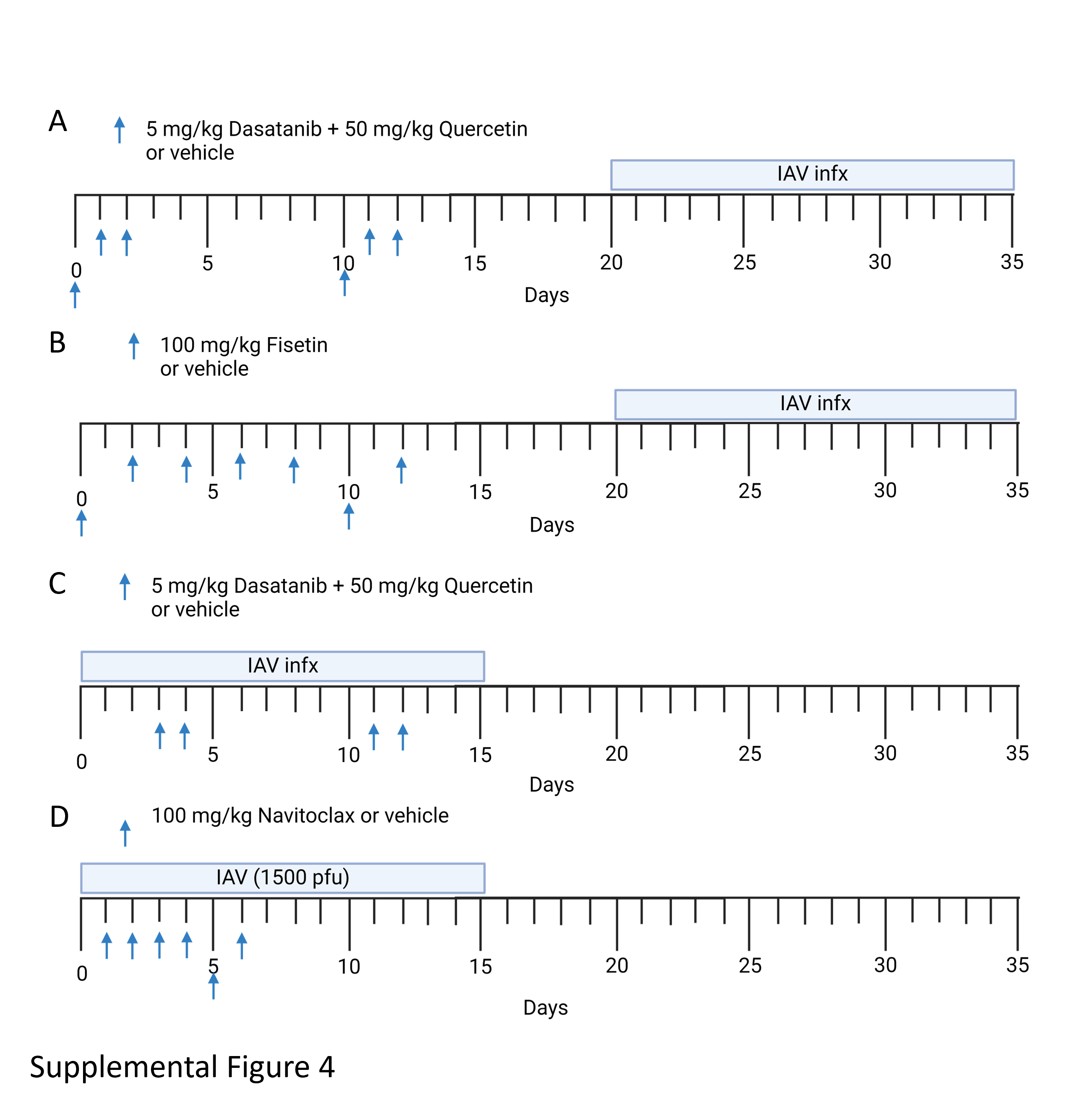

Supplement: Supplementary file 1 — Figures S1–S7. [file ACEL-24-e14437-s001.zip › Supplemental figure 4_resubmission.tif]

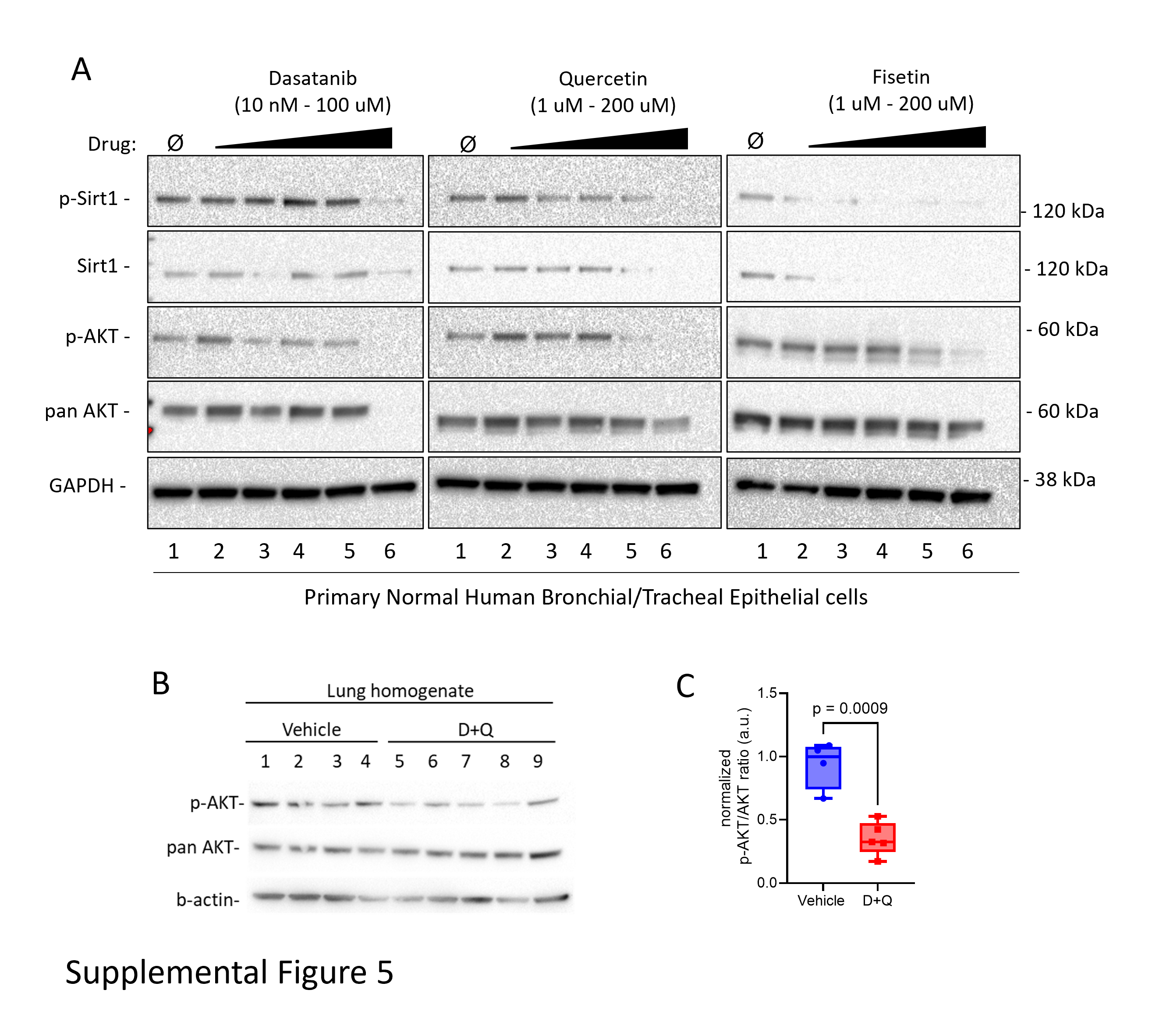

Supplement: Supplementary file 1 — Figures S1–S7. [file ACEL-24-e14437-s001.zip › Supplemental figure 5_resubmission.tif]

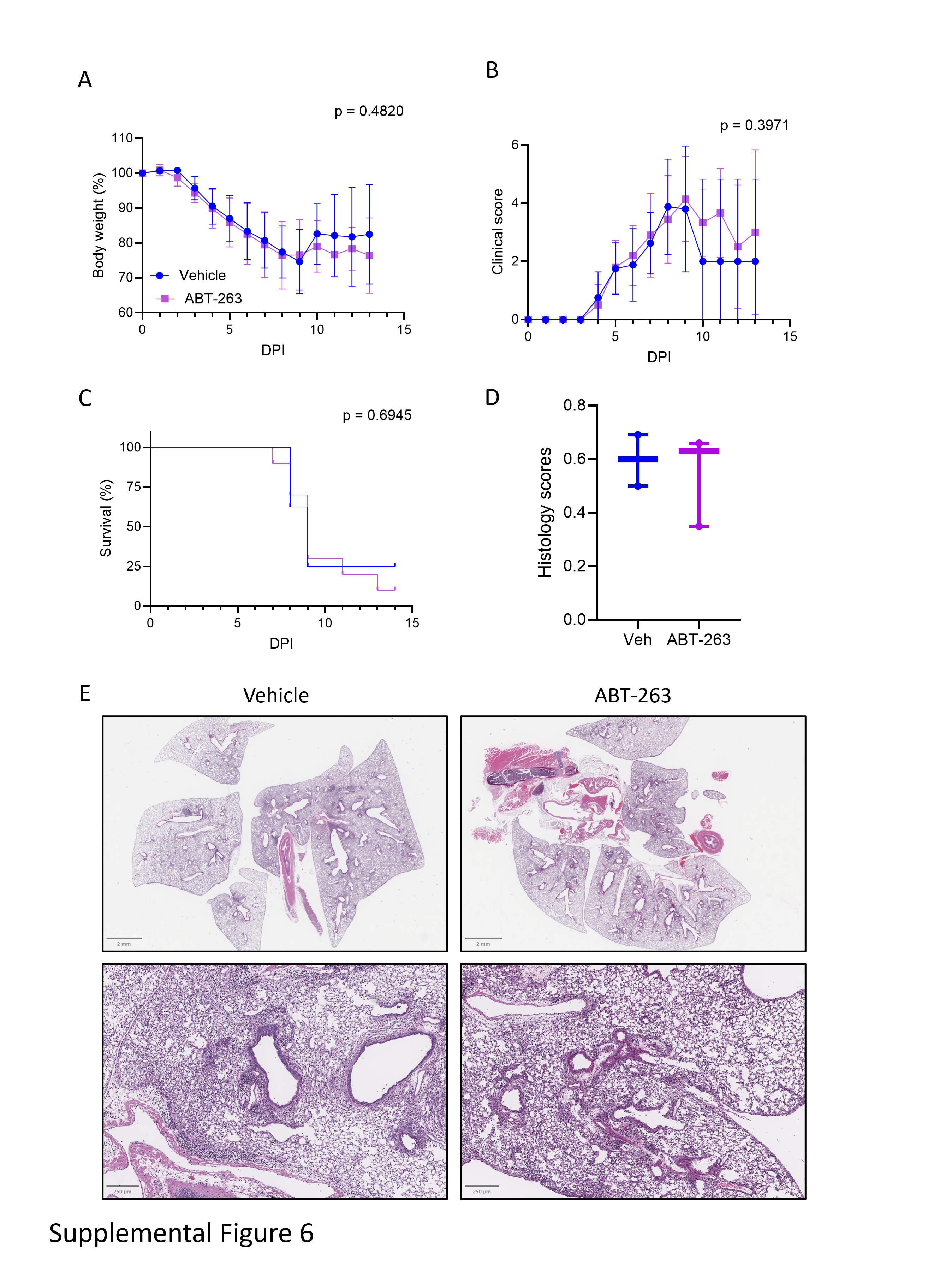

Supplement: Supplementary file 1 — Figures S1–S7. [file ACEL-24-e14437-s001.zip › Supplemental figure 6_resubmission.tif]

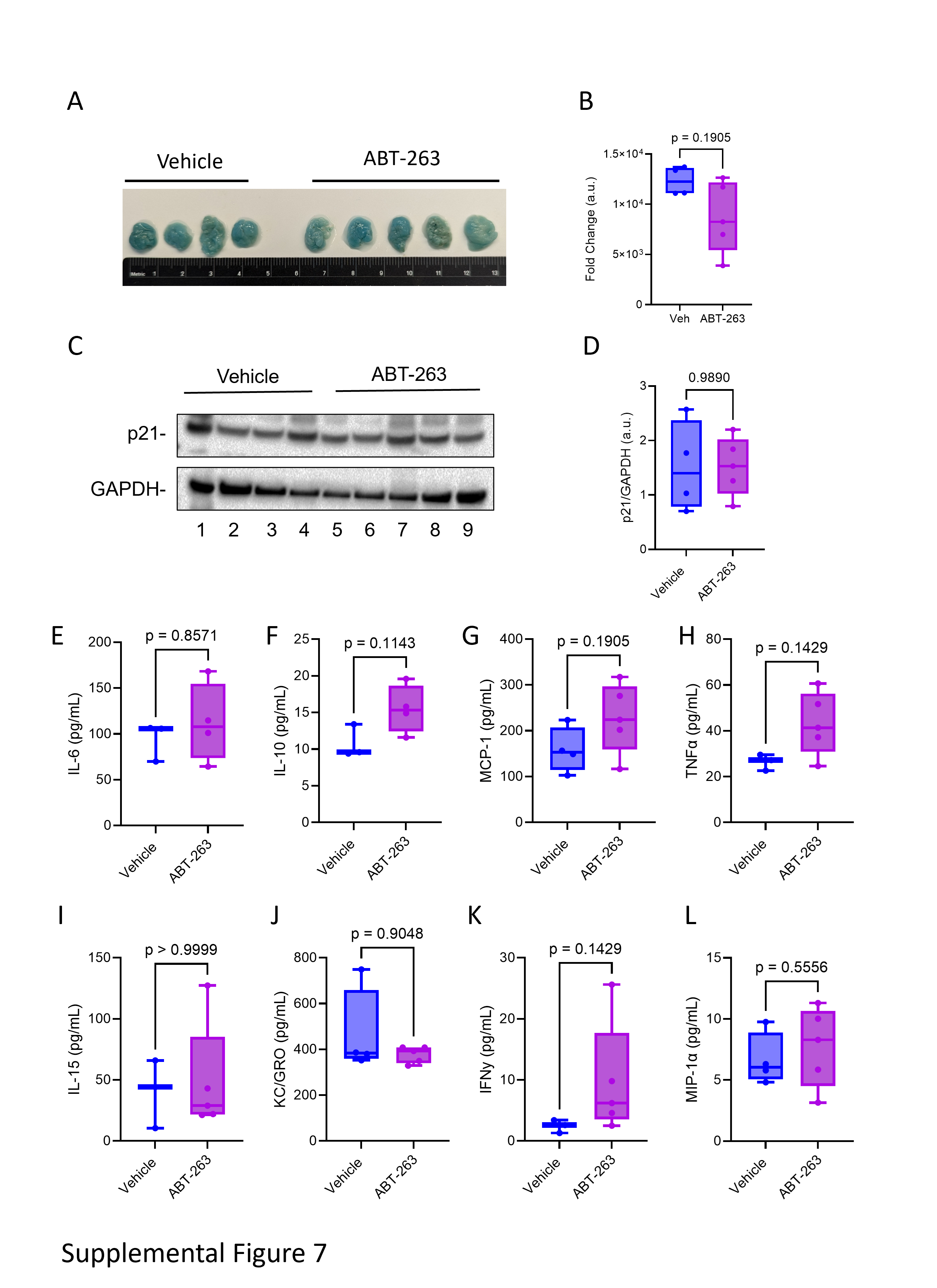

Supplement: Supplementary file 1 — Figures S1–S7. [file ACEL-24-e14437-s001.zip › Supplemental figure 7_resubmission.tif]

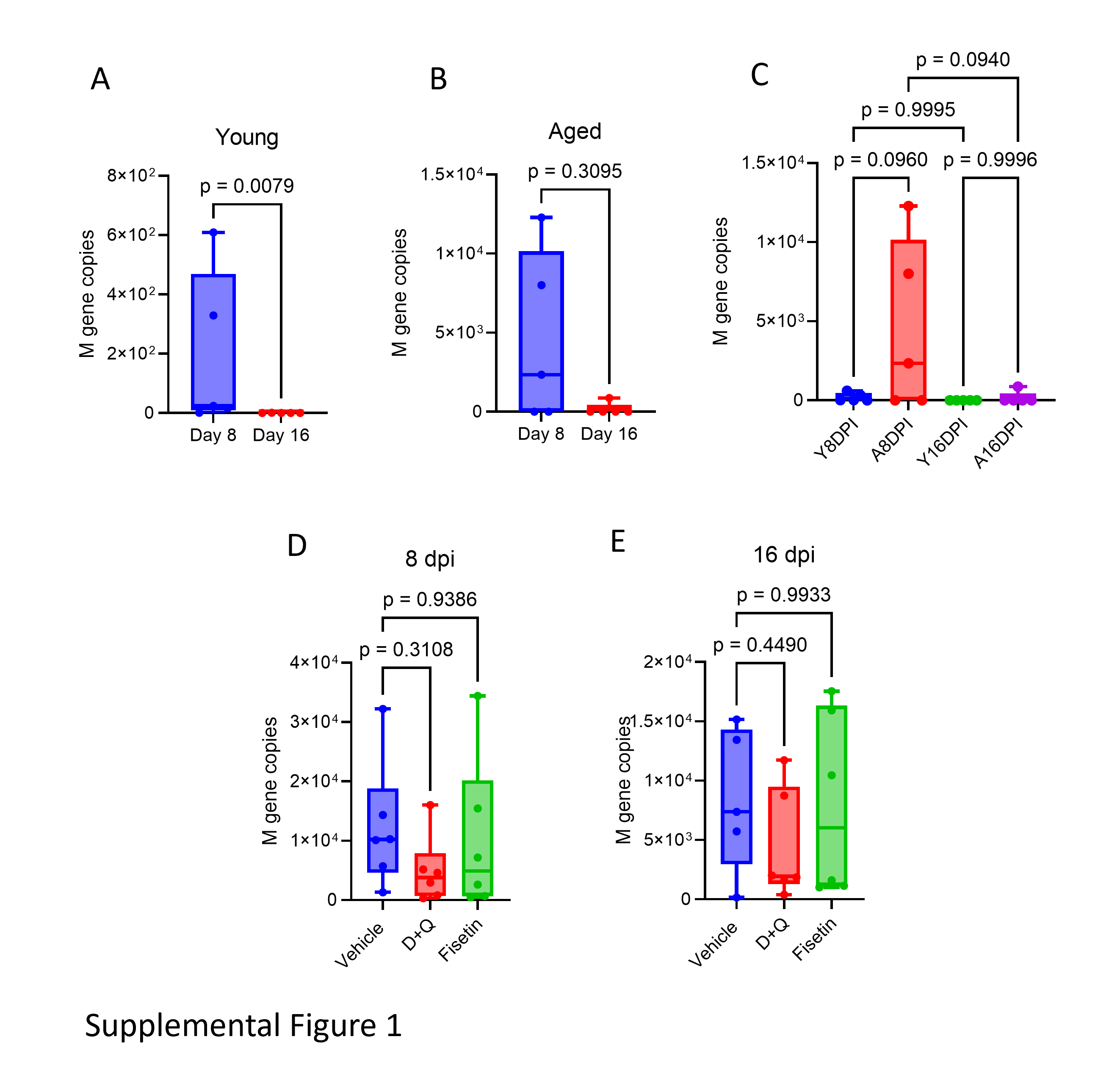

Supplement: Supplementary file 1 — Figures S1–S7. [file ACEL-24-e14437-s001.zip › Supplemental_figure_1_resubmission (2).tif]
